# Impact of Small-Scale Mining Activities on Physicochemical Properties of Soils in Dunkwa East Municipality of Ghana

**DOI:** 10.1155/2021/9915117

**Published:** 2021-11-27

**Authors:** Paul K. Essandoh, Mohammed Takase, Isaac Mbir Bryant

**Affiliations:** Department of Environmental Science, School of Biological Sciences, College of Agriculture and Natural Sciences, University of Cape Coast, Cape Coast, Ghana

## Abstract

The quality of soils in rehabilitated small-scale mined sites needs thorough investigation since a lot of changes do occur. The study assessed the impacts of small-scale mining activities on concentration and distribution of soil physicochemical properties and heavy metals. The soil samples were collected from 120 (50 m × 50 m) plots. The concentrations of soil physicochemical properties (Ca, Mg, Na, N, P, K, and OC and EC) varied significantly (*p* < 0.05) between unmined and mined soils. However, there were no statistically, significant differences (*p* < 0.05) observed in the concentrations of Cd, Hg, Pb, As, and Cu between the unmined and mined soils. Despite the generally poor (33.8%) soil quality in the study area, mining activities further reduced it by 24.2%. Soils from mined sites with unfilled/partially filled pits had higher levels of K, Mg, and Na. As mined sites fallow period increased, concentrations of OC and Cd increased, while Ca, Mg, pH, Cu, Pb, and As and value of EC decreased. The number of years that mined land remained fallow, and whether the pits were filled or unfilled during this period should be factored into the mined land rehabilitation processes.

## 1. Introduction

The gold mining industry continues to serve as a major source of revenue for government and also sustains the livelihoods of a lot of inhabitants especially in the mining communities in Ghana [1–3]. Over the years, the contribution of small-scale mining to total gold production in Ghana has been going up. From an output of 17,234 oz (3.2% of Ghana`s total production) in 1999, small-scale mining contributed 1,498,722 oz of gold forming 34.31% of the total gold production in 2014 [3]. Though small-scale gold mining continues to support growth and development of Ghana`s economy, the adverse impacts of gold mining on soil and water resources have been severally documented [4–11].

Due to the benefits of small-scale mining of gold especially to the community members, there has been an upsurge of small-scale mining activities in most communities in Ghana, and because of their large numbers, closeness of the mining sites to another and the methods used in extracting the mineral and destruction of the environment have become compounded [12] posing more difficulties in the rehabilitation of small-scale mined sites [13]. This has resulted in large tracts of unproductive and inaccessible small-scale mined sites and unhealthy competition for land resource between the miners and community members [14, 15]. Despite all these challenges, alternative livelihood strategies are not available or they are not in good standing to support the marginalized and vulnerable people, who incidentally are farmers in the municipality. Presently, mining activities and processes still occupy peripheral position on the economic ladder [16], and most of the community members without alternative sources of generating income engage in small-scale mining [17] without considering the ecological and environmental consequences. Small-scale mining is fast replacing small-holder farming as the main source of income for the local inhabitants in the study area, and it is therefore essential to help small-scale-scale miners to obtain the most benefits from mining and also provide assistance for effective rehabilitation of small-scaled mined sites for other uses. Information on physicochemical changes that occur in illegal small-scale mined soils have not been fully researched and determined.

This study assessed how small-scale mining activities influenced the level and distribution of some physicochemical properties and heavy metals in soil; how the length of fallow period after mining and the pits condition (filled and unfilled pits) affected the levels and distribution of physicochemical properties and heavy metals in soil.

## 2. Methodology

### 2.1. Study Location

The rocks in the study area have rich mineral deposits (Birimian and Tarkwaian formation) making the site attractive for gold mining, especially small-scale mining [8, 14]. The area provides a good location for the study on how the activities of small-scale mining impact on soil and ultimately on farming in the local communities. The major soils found in the study area are the Forest Ochrosols [18]. The pH of the soils is between 4.6 and 5.0. The soils are of Asikuma series belonging to the suborder Hygropeds in the Latosol soil family group. The area lies in a semideciduous forest zone with a total annual mean rainfall between 1200 mm and 2000 mm, and the mean temperature ranges from 24°C to 29°C [19].

### 2.2. Soil Sampling Locations

**Figure 1:**
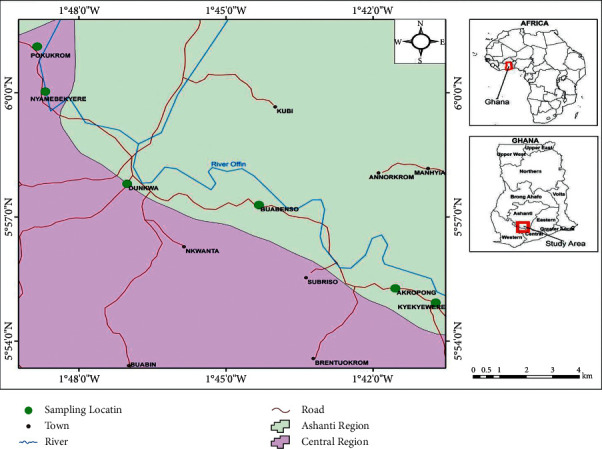
Map of study area showing the sampling towns.

The soils for the study were obtained from 14 sites in 6 towns, Akropong, Kyekyewere, Dunkwa, Nyamebekyere, Pokukrom, and Buabenso in the Dunkwa East Municipality (Figure 1). Ten sampling sites were in the mined areas (Table 1), and 4 sampling sites were in the unmined sites (Table 2). For the mined areas, 16 soil samples each from two sampling sites were obtained from Akropong, Nyamebekyere, and Dunkwa, respectively. The rest of the soil samples in the mined areas were 26 samples from 3 sampling sites in Kyekyewere and 8 samples from one sampling site in Pokukrom. Thus, a total of 82 soil samples from the mined area were used for this study (Table 1). For the unmined study area, 38 soil samples were obtained: 8 soil samples each from Akropong and Pokukrom, 15 soil samples from Buabenso, and 7 samples from Dunkwa (Table 2). In all, a total of 120 soil samples were used in the study. For the mined areas, the sampling locations were selected based on the years of fallow of the sampling site after mining and the pit status (filled or unfilled) (Table 1).

The sampling locations and number of soil samples taken from the unmined study areas have been presented in Table 2.

### 2.3. Soil Sampling

A 200 m × 200 m belt transect was constructed across each of the mined and unmined study sites. Each belt transect was divided into 16 plots with each plot measuring 50 m × 50 m. In all, 11 belt transects (200 m × 200 m) were constructed in the mined areas and 5 belt transects (200 m × 200 m) in the unmined study sites for the soil sampling. At each sampling location, 8 out of the 16 constructed plots (50 m × 50 m) were randomly selected for sampling. Soil samples were taken from the midpoint of each randomly selected plot. The soils were collected with hand auger to the depth of 0–30 cm at each sampling point. Soil replicates (three replicates for each sample) were taken to help monitor the precision of the overall procedure and field variability [20]. The soils were sealed in plastic bags and returned promptly to the laboratory and air-dried. The soils were then rolled gently with a roller, and clods were broken to facilitate drying. Soil debris and larger coarse fragments were hand-picked. Mortar and pistil were used to grind the soils to break down the soil aggregates and reduce soil particle size so as to pass through a 2 mm sieve. The soils were then screened through a 2 mm sieve using robber stopper to obtain representative subsamples [21]. The sieved soil samples were put into plastic pots and sent for analysis in the laboratories of School of Agriculture, University of Cape Coast and Soil Research Institute, Kumasi.

### 2.4. Determination of Concentrations of Soil Elements by Atomic Absorption Spectrometry

The atomic absorption spectrometry [22] was used in analysing the concentrations of most of the soil elements considered in the study. These elements included magnesium (Mg), calcium (Ca), sodium (Na), potassium (K), cadmium (Cd), mercury (Hg), copper (Cu), lead (Pb), and arsenic (As).

1.0 g of sieved soil sample was placed in a 16- by 150-mm test tube. One small Teflon-covered magnet and 5 ml concentrated nitric acid were added. The tube and contents were placed in one hole of an aluminum heating block fitted on top of hotplate equipped with a magnetic stirrer. The sample-acid mixture was agitated and heated to boil whilest continuously stirring for 30 minutes. The tube from the block was removed, and the solution was allowed to cool slightly before adding 5 ml of distilled water. The mixture was heated to boil and then allowed to cool. The mixture was made up to 10-ml mark with concentrated nitric acid. The solution was then thoroughly mixed and centrifuged (Agilent Technologies).

### 2.5. Instrumental Analyses

A Hitachi Model Z-8000, flame and graphite furnace atomic absorption spectrophotometer (Hitachi, Ltd., Tokyo, Japan) was calibrated and tested by atomizing five working standard solutions and manually measured the percent absorption as per manufacturer recommended operating conditions. Calibration solutions were freshly prepared by successive dilution of the stock standard solutions immediately before analysis. All the measurements were based on an integrated absorbance and were performed using a Zeeman-effect background correction system with respective element hollow-cathode lamps as the light sources. The concentrations of the various elements were determined by flame atomic absorption spectrometry (FAAS) technique in air-acetylene flame (2000–2300°C) [22].

The soil pH was determined by the use of a pH meter (Orion star, A211 by Thermofisher Scientific Inc., NY) as described by Summer. The bulk density of soil was determined by a method described by American Society for Testing Materials (ASTM) (1995), and the soil electrical conductivity was obtained using a conductivity meter (AD 8000 by Adwa Instruments, Hungary) in a protocol described by Korsaeth [23] and ASTM [24]. The Walkley–Black chromic acid wet oxidation method [25] was used in determining the organic carbon of soil samples, whilst the two-step digestion-UV spectrophotometry method was employed in determining soil nitrogen [26] by measuring absorbance at 275 nm and 220 nm. The Dickman and Bray`s method [27] was used to determine concentration of phosphorous in soil, and the KCL extractable acidity protocol as described by Dai and Ritcher [28] was used to determine the extractable acidity of the soil.

### 2.6. Analysis of Soil Data

The soil data obtained was subjected to both basic descriptive and detailed statistics of standard deviation, correlation, and LSD, which were achieved using Microsoft excel for data entry, SAS for mean, range, ANOVAs, and mean separation (LSD). The data for the soil samples were subjected to correlation analysis using SYSTAT version 8.0 software.

### 2.7. Determination of Soil Quality Index

The study area lies in a semideciduous forest zone, and the mineral soil property threshold levels, interpretations, and associated soil index values were obtained from the method developed by Amacher et al. [29] for forest soils. The individual index values for all the mineral soil properties measured (16 physical and chemical properties) were summed to give a total soil quality index (total SQI = Σ individual soil property index values) value of 18. The total SQI was then expressed as a percentage of the maximum possible value of the total SQI for the soil properties that were measured:

SQI (%) = (total SQI/maximum possible total SQI for properties measured) × 100.

## 3. Results

### 3.1. Soil Parameters in the Unmined and Mined Soils

The distribution of soil parameters across the unmined and mined soils were analysed, and there were significant differences (*p* < 0.05) in the Cu, EC, exchangeable acidity, OC, Mg, Na, Ca, K, P, and N, between soils of unmined and mined sites. There were higher concentrations of Cu, OC, Mg, Na, Ca, K, P, and N in the unmined soils as compared to that of the mined soils (Table 3).

The concentrations of heavy metals in the soils were generally within the permissible levels for agricultural soils except Cd in the mined soil which was higher than the prescribed EU [33] standard (Table 3).

Soil nutrients N, K, Ca, and Mg in both the unmined and mined soils were within the low ranges for agricultural soil; however, the concentration of P in the unmined soils which was within the optimal range (10.9–21.4 *µ*g/g) for agricultural soil reduced to 7.01 *µ*g/g (low level) in the mined soils (Table 3).

### 3.2. Correlation of Physicochemical Properties and Heavy Metals in Soil

Na and K (*r* = 0.866) likewise Mg and Ca (*r* = 0.706) were highly correlated (Table 4). Exchangeable acidity strongly correlated with both K (*r* = 0.576) and Na (*r* = 0.674). Other variables that were strongly correlated included Na and Ca (*r* = 0.534), P and N (*r* = 0.536) and K and N (*r* = 0.633) (Table 4).

### 3.3. Distribution of Soil Parameters across Mined Sites with Filled and Unfilled Pits

There were significant differences (*p* < 0.05) in the concentrations of Na, K, Mg, OC, EC, Cu, and Hg between soils of mined areas with filled and unfilled pits. The concentrations of Hg, EC, Mg, K, Na, and Cu were higher in the soils of mined sites with unfilled pits; and the level of OC in the soils was relatively higher in the mined sites with filled pits.

### 3.4. Soil Quality Index Scores by the Sampling Site

The result of the soil quality index (SQI) scores by site for the whole sampled area showed that, out of the maximum SQI score of 18, no sampling site had cumulative score less than 2. Similarly, no sampling site had a cumulative score of greater than 8 (Table 5).

Fifteen locations representing 12.5% had a cumulative score of 3. The maximum SQI score of 8 in this study was obtained in three locations. Thirty-four locations forming 28.3% of the sampling sites had a SQI score of 5 (Table 5). The percentage SQI of the unmined soils ranged from 16.7% to 44.4%, and that for the mined soils occurred between 16.7% and 38.9% (Table 6). There were more sampling sites with relatively higher SQI values in the unmined soils as compared to the mined soils (Table 6). The average SQI values of the unmined and mined soils were 33.8% and 24.2%, respectively.

From Table 7, OC, Ca, and N were strongly correlated with total SQI. All three correlations were statistically significant at an alpha level of 0.01. Although the correlations between total SQI and soil parameters such as Cu, Cd, and Pb were statistically significant, they were not strong.

### 3.5. Effect of Years of Fallow of the Mined Site on the Concentration of Physicochemical Properties and Heavy Metals in Soil

It is discernible from Table 8 that the number of years that mined soil laid fallow (0–5 years) does not influence Na, P, K, and Hg levels in the soil. However, the number of years that the mined soil laid fallow influenced pH, EC, and OC. Similarly, years of fallow influenced the levels of Mg, Ca, Cd, Cu, Pb, and As in the soil. The relationship between years of fallow of mined site and the following OC and Cd showed that, as the number of years that mined site remained fallow increased, the concentrations of OC and Cd increased; and in the negative relationship, the concentrations of pH, Ca, Mg, Cu, Pb, As, and EC in the soil reduced.

## 4. Discussion

### 4.1. Distribution of Heavy Metals and Physicochemical Properties in Mined and Unmined Soils

Physicochemical properties of soil affect the mobility and pathways of nutrients and pollutants, and it is therefore important to assess these soil physicochemical properties in order to determine their mutual relationships which influence the use of these soils for other activities such as sustainable agriculture [34]. The mean concentrations of Cu in the unmined and mined soils (Table 3) were below the WHO/FAO [35] prescribed value of 100 mg kg^−1^ and European Union [33] value of 50 mg kg^−1^ for agricultural purposes. The concentration of Cu in mined soil in the study area was far lower than the findings by Kpan et al. [8] who reported that the mean concentration of Cu in small-scale mined soils in some towns in the Dunkwa East Municipality was 63.26 mg kg^−1^. This study was done on illegal small-scale mining sites located deep in the forest where influence of other anthropogenic source was very minimal. Ali et al. [36] obtained Cu concentration range of 4.85–34.65 mg kg^−1^ in gold mined soils in Sudan. Gold mining activities lead to release of Cu which binds to particles of organic matter, clay materials, and sesquioxides resulting in accumulation of Cu in the mined soil [37]. The concentrations of Pb in the soils of the study area (Table 3) were lower than the WHO/FAO [35] permissible Pb levels (50.00 mg kg^−1^) in agricultural soils. The observed concentration of Pb in the soils of the area could be due to lower concentration of Pb in the parent rocks and use of fuels that do not contain Pb. The relatively higher level of Pb in mined soil (Table 3) could be that mine tailings produce acidic leachate that contain salts and trace elements which on exposure or dispersal by wind can cause increased levels of trace elements in the soil [37, 38]. In a study done by Kpan et al. [8] in soils of small-scale mining towns in the Dunkwa East Municipality of Ghana, the mean concentration of Pb was 95.13 mg kg^−1^ exceeding the WHO/FAO guidelines. The finding by Antwi-Agyei et al. [39] showed that the average Pb concentration in soils around tailing dams in Obuasi, Ghana, was 24.22 mg kg^−1^; this value was lower than the WHO/FAO standard. According to Lo et al. [40] outbreak of Pb poisoning among children in two villages in the Zamfara state in Nigeria was due to gold mining activities.

The mean concentrations of Cd in the unmined and mined soils (Table 3) were below the WHO/FAO [35] permissible limit of 3 mg kg^−1^ for agricultural soils, but that for the mined soil was greater than the threshold limit of 1.0 mg kg^−1^ for the European Union [41]. The concentrations of Cd (2.46–3.58 mg kg^−1^) in some mined sites of Nyamebekyere in this study could pose health risk as higher amounts of Cd in soils could result in relatively higher amounts of Cd in edible leaves of crop plants [42, 43]. This finding is in line with Bitala et al. [44] who obtained concentrations of Cd in mined soils ranging from 6.4 mg kg^−1^ to 11.7 mg kg^−1^ in Tanzania. Mining activities such as mineral excavation, ore transportation, smelting, and disposal of tailing and waste water lead to flow and accumulation of Cd in soil [45]. Long-term exposure to Cd through soil and food intake can cause cancer and organ system toxicity [46]. The mean concentration of As in soils of the unmined and mined areas (Table 3) was lower than the mean concentrations obtained by researchers working on gold mined soils in Wantia (23.14 mg kg^−1^) and Fel (10.73 mg kg^−1^) in Kombo-Laka in Cameroon [47]. Arsenic occurs naturally and is widely distributed in the Earth`s crust [9].

Historically, small-scale mining for gold often leads to high Hg levels in the soils of mined areas [41]. However, the concentration of Hg in the soils of the study area (Table 3) lies within the permissible limit of the WHO/FAO [35] value of 2.00 mg kg^−1^ [48]. This corroborated with work done by Basu et al. [49] who observed that some gold mined soils in Ghana had lower Hg concentration than the guideline values. The mean Hg concentration of 0.141 mg kg^−1^ obtained in small-scale mined soils in Dunkwa East Municipality by Kpan et al. [8] was higher than that of this study (0.05 mg kg^−1^). This could probably mean that the use of Hg for small-scale mining activities on the field in the study area is generally going down. However, the concentrations of mercury in soils of some of the mined areas in Kyekye were (0.191 mg kg^−1^, 0.185 mg kg^−1^, and 0.184 mg kg^−1^) closer to the WHO/FAO [35] permissible limit. Mercury is an environmental pollutant of most small-scale mining areas and can lead to serious adverse alterations in the human body tissues [49, 50].

The pH of soils in the whole study area ranged from 4.60 to 5.80 (Table 3). Soil pH is considered as a master variable influencing the chemical, physical, and biological properties of soil [31], and a pH level of 6-7 is normally suitable to maintain productivity of crops. The pH levels obtained in soils of the unmined study area ranged from 4.60 to 5.80 and that for the mined area varied from 4.60 to 5.20 (Table 3). The increase in the concentrations of Pb, Cd, and Hg in the mined soils could have influenced the relatively lower pH levels of the mined areas. The low pH of soils in both unmined and mined areas were similar to pH values (5.20–6.60) obtained by Leopold et al. [47] who studied the effects of gold mining on soils in the Fel mined area and 4.00–6.20 in Wantia mined area in Kombo–Laka Area of Cameroon. The relatively lower pH levels in the mined areas of this study collaborated with the findings by Oladipo et al. [51] that mining activities caused reduction in soil pH in the unmined areas of Awo (6.50), Itagunmodi (6.70), and Ijero-Ekiti (6.80) to 5.10, 5.30, and 3.50, respectively.

Low Na concentration in soil is considered beneficial for the growth and development of many plants [52]. Plants have different salt tolerance levels, and high concentration of Na in the soil can result in low productivity of crops and also lead to sodium dispersion making agricultural soils unsuitable for growing crops [30]. If the concentration of Na in an agricultural soil is greater than 2 cmol_c_ kg^−1^, then it is on the very high level. Lower concentration of Na ranges from 0.1 to 0.3 cmol_c_ kg^−1^, and moderate levels of Na in agricultural soils occurs between 0.3 and 0.7 cmol_c_ kg^−1^ [32]. The concentration of Na in both the mined and unmined soils (Table 3) falls within the moderate level for agricultural purposes.

High EC in agricultural soils adversely influences crop yield and soil living organisms [30, 52]. For surface soils (0–30 cm), EC values of 4–8 dSm^−1^, 8–16 dS m^−1^, and >16 dS m^−1^ are considered as moderately saline, saline, and very strongly saline, respectively [30, 52]. The EC values in the unmined soils (0–30 cm) and that of the mined soils (0–30 cm) (Table 3) are in line with work done by Leopold et al. [47] in Cameroon which showed that EC in the mined soils of Fel ranged from 0.098 to 0.257 dS m^−1^ (mean value of 0.166 dS m^−1^) and that of Wantia also ranged from 0.119 to 0.189 dS m^−1^ (mean value of 0.15 dS m^−1^). The EC values of gold mined soils in Dar-Mali locality in Sudan ranged from 0.13 to 20.9 dS m^−1^ indicating that soils in that area varies from nonsaline to extremely saline soils at different sites [36].

The relatively lower concentration of Ca in the mined soil (Table 3) could be due to small-scale mining activities in the area. Mining activities often lead to elevated levels of acid deposition and subsequent acid leaching which may increase loss of cations including Ca and Mg [6, 53]. According to Oladipo et al. [51], gold mining generally reduces the level of calcium in the soil. Due to mining activities, the concentration of calcium in soils of Itagunmodi and Ijero-Ekiti areas in Nigeria reduced from 41.82 cmol_c_ kg^−1^ to 22.48 cmol_c_ kg^−1^ and from 29.57 cmol_c_ kg^−1^ to 14.96 cmol_c_ kg^−1^, respectively. Moreover, the calcium concentration in the mined soils of this study differed significantly (*p* < 0.05) from that of the unmined soils, and this observation collaborated with work done by Oladipo et al. [51]. For agricultural soils, Ca concentration less than 5.0 cmol_c_ kg^−1^ is considered low, between 5 and 10 cmol_c_ kg^−1^ as moderate, and greater than 20 cmol_c_ kg^−1^ as very high [32].

Mg is an important element in many physiological and biological processes in plants and thus supports growth and development and also serves as defense mechanism during abiotic stress periods in plants [54]. The observed concentrations of Mg for the unmined and mined areas (Table 3) collaborated with work done by Oladipo et al. [51] that small-scale mining activities caused reduction in the concentration of Mg in soils of Awo, Itagunmodi, and Ijero-Ekiti areas in southern Nigeria. The concentrations of Mg in the soils of unmined and mined areas for this study were within the low range of 0.03 to 1.0 cmol_c_ kg^−1^ for agricultural soils [32].

Gold mining activities generally have adverse effects on the concentration of N in the soil [51]. In this work, the concentration of N in the whole study area varied from 0.01% to 0.2% (Table 3). According to Hazelton and Murphy [32], when the concentration of N in agricultural soils is between 0.05 and 0.15%, it is considered low; between 0.15 and 0.25% as medium; 0.25 to 0.50% as high; and >0.5 of nitrogen as very high level. Thus, the concentration of N in the soils of the study area was low. The significant difference (*p* < 0.05) between the nitrogen levels of the mined and unmined soils in this study could be due to small-scale mining activities as this observation was in line with the finding by Eludoyin et al. [6] who stated that small-scale mining activities caused a significant (*p* < 0.05) loss of soil nitrogen in southwestern Nigeria. Small-scale mining activities lead to low N content of mined soil due to the minimal nitrogen source and the existence of organic matter with a high C/N ratio in the mining area [55].

Soil K is related to the parent material that formed the soil and the degree of weathering [56]. K is important for growth and development in plants as it promotes movement of water and nutrients [56]. The significant difference (*p* < 0.05) between K in the unmined soil and that of the mined soil (Table 3) suggests that small-scale mining activities caused reduction of K in the mined soils probably as a result of acid leaching which promotes loss of basic cations such as K [53]. This finding is in line with observation made by Eludoyin et al. [6] that the level of K in relatively undisturbed soils (0.12 cmol_c_ kg^−1^) was higher than that of mined soils (0.07 cmol_c_ kg^−1^) in southern Nigeria. According to work done by Dorgbetor et al. [57] on the quality of mined soils in Obuasi, Ghana, the level of K in the unmined soils (0.16 cmol_c_ kg^−1^) got reduced in the mined soils (0.10 cmol_c_ kg^−1^). Agricultural soils with K concentrations between 0.128 and 0.256 cmol_c_ kg^−1^ are considered to have low level of K, and when the level of K ranges from 0.256 to 0.641 cmol_c_ kg^−1^, it is considered adequate [32].

The concentration of P in the mined soils and that of the unmined soils (Table 3) observed in this study corroborated with work done by Oladipo et al. [51] who reported that gold mining activities reduced the levels of available P in unmined soils of Awo (101.04 *µ*g g^−1^), Itagunmodi (78.89 *µ*g g^−1^), and Ijero-Ekiti (60.50 *µ*g ^−1^g) to 86.64 *µ*g ^−1^g (Awo), 53.90 *µ*g g^−1^ (Itagunmodi), and 49.13 *µ*g \ g^−1^ (Ijero-Ekiti), respectively. The concentration of P in the mined soils in the present study was lower than the prescribed level of P for agricultural soils which ranges from 25 to 35 *µ*g g^−1^ [32]. However, the mean concentration of P in the unmined soils was within the optimal range of 10.9 to 21.4 *µ*g g^−1^ for agricultural soils. According to Salem et al. [58], mining activities often cause reduction in fungi needed to mine the soil for P, and relatively lower levels of pH and organic matter in mined soils lead to reduced P content in the soil.

Soil OC is vital for the biological, chemical, and physical functioning of agricultural soils [32]. The concentration of OC in the soils of the study area varied from 0.19 to 3.60%. The concentration of OC obtained in the mined area (Table 3) is within the low-level range of 0.4 to 1.0% prescribed by Hazelton and Murphy [32] for agricultural soils. However, the concentration of OC in the unmined area (Table 3) falls within the moderate level of 1.0 to 1.8% for agricultural soils. In evaluating the quality of mined soils in Obuasi, Ghana, Dorgbetor et al. [57] observed that gold mining activities caused reduction of OC in mined soils. Gold mining activities often lead to destruction of all vegetation of organic material resulting in OC decrease in the mined soil [55]. Although the concentrations of Na, Ca, Mg, N, P, and K and value of EC in the soils of the study area were generally below the prescribed agricultural levels [32], small-scale gold mining activities further reduced their levels in the mined soils. Oladipo et al. [51] noted in south western Nigeria that soil properties such as N, P, and EC and exchangeable cations (Ca, K, Na, and Mg) differed significantly (*p* < 0.05) between the soils of mined and unmined (control) areas.

### 4.2. Distribution of SQI and Physicochemical Properties in the Study Area

In this study, the unmined and mined soils had an average SQI value of 33.8% and 24.2%, respectively (Table 6). The SQI value of the unmined soil (33.8%) suggests that the poor quality of the soil of 66.2% relative to the optimum quality was due to the inherent poor soil properties [57]. Though the poor quality of the unmined soil is an inherent characteristic, the impact of small-scale mining activities on the soil further widened the difference in quality between the maximum obtainable (SQI value of 18) and the inherent quality as was reflected in the lower SQI values for soils in the mined areas (Table 6). According to Asensio et al. [59], the quality of soils in gold mined areas tend to be low. In this study, the SQI for the mined soils was 9.6% less than the SQI value for the unmined soils. This is in line with the work done by [57] on gold mined soils in Obuasi, where they observed an SQI of the mined soils of 12.1% less than that of the unmined soils.

The Pearson's correlation coefficient matrix for physicochemical variables and total SQI of the soil samples (Table 7) indicated a very strong correlation between OC and total SQI (*r* = 0.584; *p* < 0.01), Ca and total SQI (*r* = 0.522; *p* < 0.01), and N and total SQI (*r* = 0.535; *p* < 0.01). These strong positive correlations may indicate that, in about 58.4%, 52.2%, and 53.5% cases, the levels of OC, Ca, and N in the soil were observed to have increased with increase in SQI values and vice versa, respectively.

### 4.3. Effect of Years of Fallow of Mined Site on the Concentrations of Physicochemical Properties and Heavy Metals in Soil

OC improved with years of fallow, and this could be as a result of the high biomass production and decay of organic matter or leaf litter accumulation over time. Costa and La Mantia [60] reported an accumulation of litter and a subsequent increase in the carbon content of the soil following the processes of abandonment. This is evidenced by the lowest OC content recorded in the newly abandoned small-scale mine sites. Some of these sites at the study area had a bare soil surface with no tree cover and hence had little or no accumulation of organic matter. This condition, however, creates a hostile environment where natural successional processes leading to the establishment of vegetation cover is much slower for the least fallow soils.

The elevated levels of Cd in the soils of higher fallow years as compared to the soils of the lower fallow years could be due to the geochemical composition of the parent material and Cd inputs from diffuse anthropogenic sources such as atmospheric deposition [61]. The bioavailability of Cd is very high as compared to other heavy metals due to it higher solubility and low energy binding to soil component. Lower pH in soils increases the mobility and bioavailability of Cd in soils. Additionally, transformation of Cd from an immobile form to an easily bioavailable form is enhanced by acidic soils [61]. As observed in this study, as the fallow years increase, pH reduces and Cd levels increase.

The comparatively lower pH content for the older fallow sites could be attributed to acidic parent materials such as pyrites and rainfall-associated leaching. Pyrite parent materials, which are predominant in the study area, may oxidize to sulphuric acid and significantly lower soil pH [62]. The higher pH recorded for the younger fallow sites could be due to carbonate bearing minerals (Ca/MgCaCO_3_) which tend to increase and buffer pH as they weather and dissolve [63]. Elevation of organic matter decomposition on degraded soil increases soil pH; however, soil pH decreases with the length of time after restoration, and this is because of litter inputs and exudates from roots and microbes [64].

The EC results indicate that the older reclaimed sites had high water-soluble physicochemical elements available for plant uptake because EC has been correlated to concentrations of nitrates, K, Na, chlorides, ammonia etc., in the soil [62, 64]. These sites, for example, were enriched with organic matter, which improves soil water holding capacity and cation exchange than the younger reclaimed site. High levels of precipitation could flush soluble salts out of the younger reclaimed sites, which had no or limited vegetative cover and hence low conductivity levels over time in soils of higher fallow years.

The availability of Mg in the soil depends on factors such as the distribution and chemical properties of the source rock material and its grade of weathering and site-specific climatic and anthropogenic factors. Mg bound in the interlayers of silicates is not mobile and is only released into mobile fractions through weathering processes, which is regarded as a long-termed, slow process. The reduced concentration of Ca and Mg in mined soils with longer years of fallow could be due to uptake by the roots of the growing vegetation. Also, acidic soils promote leaching of Mg and Ca [65, 66].

The reduction in Cu concentrations as the years of fallow increased could be attributed to the uptake by the growing vegetation on the fallow soils. Additionally, rainfall could also facilitate the translocation of Cu into deeper depths in the soil [67].

The Pb in the soil of the mined site was found to have decreased with the longer years of fallow sites. This is in support of Rodríguez et al. [68] that acidic mined soils are contaminated with heavy metals; hence, the reduction in concentrations of the heavy metals in the soils as years of fallow increased with decreasing pH. Sauve et al. [69] have also reported that low pH in mining soils increased the availability of heavy metal such as Pb in the soil.

The high As contents of the least fallow soils may probably be due to the parent material. This assertion has earlier been stated by Kwon and Lee [70] and Bowel et al. [71] that soil arsenic may be controlled by the lithology of the parent rock materials, weathering history, transport, biological activity, and precipitation.

### 4.4. Distribution of Soil Properties across Mined Sites with Filled and Unfilled Pits

The distribution of soil parameters across mined sites with filled and unfilled pits indicated that there were significant differences (*p* < 0.05) in the distribution of Na, K, Mg, OC, EC, Cu, and Hg. Concentration of Na significantly decreased in the soils of mined sites with filled pits as compared to those with unfilled pits. This difference could be as a result of filling the pits with washed tailings. Washing of the tailings removes significant amount of physical and chemical properties of the soil [72]. Additionally, pilling of the tailings prior to filling enhances leaching of Na.

Concentrations of Mg and K were higher in soils of the unfilled pits as compared to the filled pits. It was observed that the material used for filling the pits were mainly washed tailings. Washing of tailings, however, leads to a substantial loss of physicochemical properties either through leaching to deeper levels or as run off since tailings are loose soil particles [65, 66]. OC concentration was lower in the unfilled pits as compared to the filled pits. This could be as a result of the high biomass production and decay of organic matter or leaf litter accumulation after the establishment of vegetation cover. Accumulation of litter after filling and abandoning the pits correspond to an increase of the carbon storage in soils [73]. The soils of unfilled pits, however, recorded lower concentrations of OC because sampled soils from the pit could be top soil eroded into the pit or breaches from the walls in the pit.

EC of the soils of the unfilled pits were higher than that of the filled pits. The lower values recorded in the filled pits could emanate from the tailings used for the filling as washed tailings lose soluble physicochemical properties and salts. Concentrations of Cu in the unfilled pits were higher than the concentrations in the filled pits. With or without sorbing solutes, Cu could be leached into deeper layers or depth and groundwater in loose soils like the tailings used in filling the pits. Cu could also be absorbed by the growing vegetation on the filled pits [47].

Hg concentrations in the unfilled pits were higher than the concentrations in the filled pits. Higher mercury concentrations in the unfilled pits could arise as a result of the onsite use of mercury for amalgamation in small-scale mining [15]. The low concentrations recorded in the filled pits could result from the absorption of Hg by plants in the soil. Hg in the filled pits could also be converted into methyl mercury by bacteria in the soil. Rainfall could also translocate Hg into deeper depth since tailings are not compact enough to hold water.

## 5. Conclusion

Small-scale mining in the study area has affected the quality of the soil supporting vegetation growth. The mined soils are losing essential physicochemical elements that are required for plant growth and establishment. The concentrations of important physicochemical properties such as K, P, Ca, Mg, Na, and OC and the value of EC in the soils have been significantly reduced as a result of the small-scale mining activities. Within the study area, anthropogenic activities did not cause significant variation in the concentrations of heavy metals such as Cu, As, Hg, Pb, and Cd between unmined and mined soils; however, concentrations of some of the heavy metals increased in the mined area. The soil material used in refilling (back filling) of mined pits, as part of mined land restoration process, influence the quality of mined soil as soils from mined sites with unfilled/partially filled pits tend to have higher levels of important physicochemical properties such as K, Mg, and Na. The number of years that mined land remained fallow influenced the quality of soil as increase in fallow years increases the concentrations of OC and Cd. However, as the fallow years increased the concentrations of Ca, Mg, pH, Cu, Pb, and As and value of EC in the mined soil decreased. The average difference in quality between the unmined (33.8%) and mined (24.2%) soils was 9.6%.

## Figures and Tables

**Table 1:** Sampling location, pit status of sampling site, and years of fallow of the mined area.

Sample location	Year(s) of fallow	Pit status of sampling site	Number of soil samples
Akropong (1)	1	Filled	8
Akropong (2)	1	Not filled	8
Kyekyewere (1)	2	Filled	8
Kyekyewere (2)	2	Not filled	8
Kyekyewere (3)	3	Filled	10
Dunkwa (1)	4	Not filled	8
Dunkwa (2)	4	Filled	8
Nyamebekyere (1)	5	Filled	8
Nyamebekyere (2)	5	Not filled	8
Pokukrom	Freshly mined	Not filled	8
Total			**82**

**Table 2:** Sampling locations and number of soil samples from the unmined study area.

Sampling location	Number of soil samples
Akropong	8
Buabenso	15
Dunkwa	7
Pokukrom	8
Total	**38**

**Table 3:** Distribution of heavy metals and physicochemical parameters in soils of the study area.

Parameter	Unmined soil	Mined soil	Agricultural standards
Copper (mgkg^−1^)	1.4 ± 0.14	1.12 ± 0.06	WHO/FAO (2001) = 100; EU (2002) = 50
Lead (mgkg^−1^)	1.23 ± 0.02	1.28 ± 0.04	WHO/FAO (2001) = 50
Cadmium (mgkg^−1^)	0.56 ± 0.05	1.87 ± 0.13	WHO/FAO (2001) = 3; EU (2002) = 1
Arsenic (mgkg^−1^)	0.11 ± 0.01	0.08 ± 0.10	EC (2002) = 20;
Mercury (mgkg^−1^)	0.04 ± 0.01	0.05 ± 0.01	WHO/FAO (2001) = 2
Nitrogen (%)	0.15 ± 0.03	0.04 ± 0.02	0.05–0.15 = low; 0.15–0.25 = moderate, 0.25–0.50 = high
Potassium (mgkg^−1^)	0.12 ± 0.01	0.05 ± 0.01	0.128–0.256 = low 0.256–0.641 = moderate
Phosphorus (*µ*g/g)	25.86 ± 7.80	7.01 ± 4.17	25–35 = prescribed level; 10.9–21.4 = optimal level
Organic carbon (%)	1.44 ± 0.42	0.74 ± 0.23	0.4–1.0 = low; 1.0–1.8 = moderate
Sodium (cmol_c_ kg^−1^)	0.99 ± 0.32	0.46 ± 0.24	0.3–0.7 = suitable; > 2 = high
Calcium (cmol_c_ kg^−1^)	4.87 ± 1.74	1.35 ± 0.22	<5 = low; 5–10 = moderate
Magnesium (cmol_c_ kg^−1^)	0.39 ± 0.11	0.16 ± 0.01	0.03–1.0 = low
Electrical conductivity (dSm^−1^)	0.03 ± 0.01	0.03 ± 0.02	4–8 = moderately saline; 8–16 = saline; >16 = strongly saline [30]
pH	4.60–5.80	4.60–5.20	6–7 [31]

Sources of Agricultural Standards not stated on table are from Hazelton and Murphy [32].

**Table 4:** Pearson product moment correlation coefficients of physicochemical properties and heavy metals in soil.

Pearson product moment correlation coefficient															
	Na	N	P	K	Ca	Mg	OC	EXCH acid	pH	EC	Cu	Cd	Pb	Hg	As
Na	1														
N	**0.730** ^*∗∗*^	1													
P	0.294^*∗∗*^	**0.536** ^*∗∗*^	1												
K	**0.866** ^*∗∗*^	**0.633** ^*∗∗*^	0.227^*∗*^	1											
Ca	**0.534** ^*∗∗*^	**0.562** ^*∗∗*^	0.337^*∗∗*^	0.538^*∗∗*^	1										
Mg	0.407^*∗∗*^	0.426^*∗∗*^	0.133	0.414^*∗∗*^	**0.706** ^*∗∗*^	1									
OC	0.307^*∗∗*^	0.433^*∗∗*^	0.326^*∗∗*^	0.309^*∗∗*^	0.072	0.045	1								
EXCH ACID	**0.674** ^*∗∗*^	**0.554** ^*∗∗*^	0.144	**0.576** ^*∗∗*^	0.471^*∗∗*^	0.415^*∗∗*^	0.169	1							
pH	−0.014	0.011	0.153	−0.019	0.016	−0.117	0.021	−0.125	1						
EC	0.134	0.149	0.071	0.126	0.333^*∗∗*^	0.327^*∗∗*^	−0.019	0.083	0.226^*∗*^	1					
Cu	0.287^*∗∗*^	0.286^*∗∗*^	0.190^*∗*^	0.340^*∗∗*^	0.487^*∗∗*^	0.344^*∗∗*^	−0.212^*∗*^	0.294^*∗∗*^	−0.059	0.215^*∗*^	1				
Cd	−0.281^*∗∗*^	−0.264^*∗∗*^	−0.109	−0.271^*∗∗*^	−0.254^*∗∗*^	−0.218^*∗*^	−0.010	−0.137	−0.050	−0.146	−0.333^*∗∗*^	1			
Pb	−0.150	−0.003	−0.028	−0.048	0.016	−0.032	−0.155	−0.140	0.179	−0.118	0.234^*∗∗*^	−0.152	1		
Hg	0.050	−0.050	−0.164	0.120	0.170	0.173	−0.218^*∗*^	0.072	−0.097	0.004	0.190^*∗*^	−0.115	0.042	1	
As	0.077	0.175	0.109	0.102	0.333^*∗∗*^	0.189^*∗*^	−0.177	0.194^*∗*^	−0.079	0.113	0.263^*∗∗*^	−0.082	−0.012	0.249^*∗∗*^	1

^*∗∗*^Correlation is significant at the 0.01 level (2-tailed).^*∗*^Correlation is significant at the 0.05 level (2-tailed).

**Table 5:** Soil quality index of soil samples.

SQI score	Number of samples	Percent
**2**	2	1.7
**3**	15	12.5
**4**	29	24.2
**5**	34	28.3
**6**	25	20.8
**7**	12	10.0
**8**	3	2.5
Total	**120**	**100.0**

**Table 6:** Percentage SQI of unmined and mined soils.

Location	Number of sites	Percentage SQI
Unmined Pokukrom	3	33.3
	3	38.9
	2	44.4
Unmined dunkwa	4	27.8
	1	16.7
	2	33.3
Unmined buabenso	8	27.8
	6	33.3
	1	44.4
Unmined akropong	7	33.3
	1	38.9
Mined dunkwa	2	38.9
	3	33.3
	7	27.8
	2	22.2
	2	16.7
Mined Pokukrom	1	11.1
	3	16.7
	4	22.2
Mined nyamebekyere	11	22.2
	5	16.7
Mined kyekyewere	6	16.7
	9	22.2
	8	27.8
	3	33.3
Mined akropong	2	22.2
	7	27.8
	7	33.3

**Table 7:** Correlations of SQI and soil parameters (*n* = 120).


	Total SQI	N	Ca	OC	pH	Cu	Cd	Pb
Total SQI		**0.535** ^*∗∗*^	**0.552** ^*∗∗*^	**0.584** ^*∗∗*^	0 .095	0.218^*∗*^	0.370^*∗∗*^	0.354^*∗∗*^
N	**0.535** ^*∗∗*^	1	0.444^*∗∗*^	0.393^*∗∗*^	0.247^*∗∗*^	−0.139	0.064	−0.129
Ca	**0.552** ^*∗∗*^	0.444^*∗∗*^	1	0.225^*∗*^	0.237^*∗∗*^	−0.352^*∗∗*^	0.093	−0.121
OC	**0.584** ^*∗∗*^	0.393^*∗∗*^	0.225^*∗*^	1	−0.202^*∗*^	0.048	−0.012	0.083
pH	0.095	0.247^*∗∗*^	0.237^*∗∗*^	−0.202^*∗*^	1	0 .009	−0.043	−0.187^*∗*^
Cu	0.218^*∗*^	0.139	0.352^*∗∗*^	0 .048	0.009	1	−0.167	0.201^*∗*^
Cd	0.370^*∗∗*^	0.064	0.093	−0.012	−0.043	−0.167	1	0.024
Pb	0.354^*∗∗*^	−0.129	−0.121	0 .083	−0.187^*∗*^	0.201^*∗*^	0.024	1

^*∗∗*^Correlation is significant at the 0.01 level (2-tailed).^*∗*^Correlation is significant at the 0.05 level (2-tailed).

**Table 8:** Influence of fallow years of mined land on soil properties.

Variable	Coef.	SE	*p* value	95%	CI
pH	−0.04417	0.013502	**0.002**	−0.07104	−0.0173
Na	−0.02671	0.014077	0.061	−0.05472	0.001308
P	0.176907	0.481164	0.714	−0.78064	1.134454
K	−0.0029	0.002054	0.161	−0.00699	0.001185
Ca	−0.25781	0.07723	**0.001**	−0.41151	−0.10412
Mg	−0.0178	0.00816	**0.032**	−0.03404	−0.00156
OC	0.071987	0.021065	**0.001**	0.030066	0.113907
EC	−0.00547	0.001606	**0.001**	−0.00867	−0.00228
Cu	−0.10087	0.029141	**0.001**	−0.15887	−0.04288
Cd	0.757246	0.193625	**0.000**	0.371921	1.142572
Pb	−0.23195	0.061218	**0.000**	−0.35378	−0.11013
Hg	−0.00198	0.003865	0.610	−0.00967	0.005714
As	−0.01998	0.004448	**0.000**	−0.02883	−0.01112

## Data Availability

The data used to support the findings of the study are available within the article.
